# Discovery and Validation of a Prostate Cancer Genomic Classifier that Predicts Early Metastasis Following Radical Prostatectomy

**DOI:** 10.1371/journal.pone.0066855

**Published:** 2013-06-24

**Authors:** Nicholas Erho, Anamaria Crisan, Ismael A. Vergara, Anirban P. Mitra, Mercedeh Ghadessi, Christine Buerki, Eric J. Bergstralh, Thomas Kollmeyer, Stephanie Fink, Zaid Haddad, Benedikt Zimmermann, Thomas Sierocinski, Karla V. Ballman, Timothy J. Triche, Peter C. Black, R. Jeffrey Karnes, George Klee, Elai Davicioni, Robert B. Jenkins

**Affiliations:** 1 Research and Development, GenomeDx Biosciences, Vancouver, British Columbia, Canada; 2 Department of Pathology and Laboratory Medicine, University of Southern California, Los Angeles, California, United States of America; 3 Department of Health Sciences Research, Mayo Clinic, Rochester, Minnesota, United States of America; 4 Department of Pathology and Laboratory Medicine, Mayo Clinic, Rochester, Minnesota, United States of America; 5 Department of Urology, University of British Columbia, Vancouver, British Columbia, Canada; 6 Department of Urology, Mayo Clinic, Rochester, Minnesota, United States of America; Baylor College of Medicine, United States of America

## Abstract

**Purpose:**

Clinicopathologic features and biochemical recurrence are sensitive, but not specific, predictors of metastatic disease and lethal prostate cancer. We hypothesize that a genomic expression signature detected in the primary tumor represents true biological potential of aggressive disease and provides improved prediction of early prostate cancer metastasis.

**Methods:**

A nested case-control design was used to select 639 patients from the Mayo Clinic tumor registry who underwent radical prostatectomy between 1987 and 2001. A genomic classifier (GC) was developed by modeling differential RNA expression using 1.4 million feature high-density expression arrays of men enriched for rising PSA after prostatectomy, including 213 who experienced early clinical metastasis after biochemical recurrence. A training set was used to develop a random forest classifier of 22 markers to predict for cases - men with early clinical metastasis after rising PSA. Performance of GC was compared to prognostic factors such as Gleason score and previous gene expression signatures in a withheld validation set.

**Results:**

Expression profiles were generated from 545 unique patient samples, with median follow-up of 16.9 years. GC achieved an area under the receiver operating characteristic curve of 0.75 (0.67–0.83) in validation, outperforming clinical variables and gene signatures. GC was the only significant prognostic factor in multivariable analyses. Within Gleason score groups, cases with high GC scores experienced earlier death from prostate cancer and reduced overall survival. The markers in the classifier were found to be associated with a number of key biological processes in prostate cancer metastatic disease progression.

**Conclusion:**

A genomic classifier was developed and validated in a large patient cohort enriched with prostate cancer metastasis patients and a rising PSA that went on to experience metastatic disease. This early metastasis prediction model based on genomic expression in the primary tumor may be useful for identification of aggressive prostate cancer.

## Introduction

Over 240,000 men are diagnosed with prostate cancer in the U.S. annually, and a majority of them harbor local or regional disease where the long-term prognosis is excellent [1]. About half of these men undergo radical prostatectomy (RP) and nearly 40% will present with one or more clinicopathologic features such as high Gleason score (GS), extra-capsular extension (ECE), positive surgical margins (SM+), seminal vesicle invasion (SVI) or lymph node involvement (N+) that are associated with increased risk of clinical metastasis [2]–[4]. Although only a minority of these men are truly at risk of dying of their cancer [5], many of these "clinically high risk" patients will receive additional postoperative interventions (e.g., adjuvant radiation) and often suffer treatment morbidity. Conversely, many men present without adverse clinical features and yet die of prostate cancer. Current tools have limited capacity to identify, at time of RP, men that are most at risk of metastasis and prostate cancer death - such patients are currently treated aggressively only after the observation of rising PSA (Prostate Specific Antigen) or biochemical recurrence (BCR). Recent clinical trials suggest that these patients would likely have more favorable outcomes if treated earlier post-RP [6]–[10]. Thus, the limited performance of clinical factors for predicting men at highest risk for metastasis leads to suboptimal patient management.

Over the last decade, many studies have tried to address the unmet clinical need for predicting aggressive prostate cancer using individual biomarkers or gene expression signatures [11]–[30]. However, these prior efforts have not seen widespread implementation in clinical practice because none have convincingly demonstrated improved prediction over established clinical factors such as the GS. This is mainly due to limitations in sample size and power, the lengthy clinical follow up required to observe metastatic or lethal prostate cancer events and the use of BCR as a surrogate endpoint; a sensitive, but non-specific, predictor of disease progression [31]. Thus, most biomarker studies poorly sample clinically-proven aggressive prostate cancer cases. In addition, most gene expression signatures were developed with assays that required fresh or frozen tissue, which is not routinely available in clinical practice, and were limited to profiling protein-coding genes - examining only a minority of the active genome (i.e., transcriptome). In a previous report we obtained archived formalin-fixed paraffin embedded (FFPE) primary prostate cancer specimens from the Mayo Clinic tumor registry that included a large number of patients that developed metastatic disease. With long-term follow up we ascertained a biomarker signature that could identify men at risk of progression to clinical metastasis and lethal prostate cancer [20]. However, in validation we did not demonstrate a significant improvement on performance in comparison to clinical variables and we hypothesized that this may be due to the limited focus on a set of about 1,000 protein-coding genes.

Here we expand upon this work by re-profiling the patients of the original study utilizing a high-density transcriptome-wide microarray that assesses the expression of over 1.4 million RNA features including the ∼22,000 known protein-coding genes as well as many thousands of non-coding RNAs. Such non-coding RNAs are now recognized for their ability to regulate the activity of oncogenes and tumor suppressor genes involved in the development of disease recurrence and metastatic progression [32], [33]. We present the development and validation of a genomic classifier (GC) for risk prediction of early clinical metastasis that is enriched in non-coding RNAs. We demonstrate that GC provides independent and statistically significant prognostic information beyond clinicopathologic variables and show that GC outperforms previously reported gene signatures.

## Materials and Methods

### Patient Population and Clinical Outcomes

Patients from this study were selected using a nested case-control design from the Mayo Clinic Radical Prostatectomy Tumor Registry, as described previously [20]. In brief, patients that received radical prostatectomy (RP) for primary prostatic adenocarcinoma as first line treatment at the Mayo Clinic Comprehensive Cancer Center between 1987 and 2001 were retrospectively classified into the following outcome groups:

- *No evidence of disease (NED) progression group*: Exhibited no biochemical or other clinical signs of disease progression following RP, with at least 7 years of follow-up.
- *Prostate-specific antigen (PSA)-recurrence group*: Experienced biochemical recurrence (BCR), defined as two successive increases in PSA measurements above 0.02 ng/mL (with the subsequent measure 0.05 ng/mL above the first measurement) with no detectable clinical metastasis (see below) within 5 years of BCR.
- *Clinical metastasis group (metastasis)*: Experienced BCR and developed regional or distant metastases, confirmed by bone or CT scan, within 5 years of BCR. This group was referred to as Systemic Progression (*SYS*) in our previous study [20].

A total of 213 patients met the definition of metastasis group and were designated as cases [20]. For each case, one patient each from PSA and NED groups were selected based on the matching criteria described previously [20] and were designated as controls.

#### Ethics statement

This study was approved by the Institutional Review Board of Mayo Clinic and due to the archival nature of the specimens, patient consent was waived by the board.

### RNA Extraction and Microarray Hybridization

From the original study (n = 639), RNA was available for microarray from 545 unique patients. As previously described, after histopathological re-review by an expert genitourinary pathologist, tumor was macrodissected from surrounding stroma from 3–4 10 µm tissue sections from the primary Gleason grade of the index lesion (the highest pathologic GS) for total RNA extraction [20]. Total RNA was subjected to amplification using the WT-Ovation FFPE v2 kit together with the Exon Module (NuGen, San Carlos, CA) according to the manufacturer’s recommendations with minor modifications. Amplified products were fragmented and labeled using the Encore Biotin Module (NuGen, San Carlos, CA) and hybridized to Human Exon 1.0 ST GeneChips (Affymetrix, Santa Clara, CA) following manufacturer’s recommendations. Human Exon GeneChips profile coding and non-coding regions of the transcriptome using approximately 1.4 million probe selection regions (PSRs), hereinafter referred to as features.

### Microarray Processing

#### Microarray quality control

Of the 545 patients with available tissue and RNA, a total of 59 samples failed initial QC (as assessed by Affymetrix Power Tools AUC metric [34]) and were re-run. Additionally, a PC3 cell line (ATCC, Manassas, VA) control was run with each batch and used to identify unreliable features (see below). The Human Exon array data corresponding to this study are available from the National Center for Biotechnology Information’s Gene Expression Omnibus database (GSE46691).

### Microarray Normalization, Removal of Unreliable Features and Batch Effect Correction

Feature summarization and normalization of expression values were performed by frozen robust multi-array analysis (fRMA; [35]), which is available through Bioconductor. A custom set of frozen vectors were generated by randomly selecting 15 arrays from each of the 19 batches across the whole study. Features interrogated with fewer than four probes or any cross-hybridizing probes (as defined by Affymetrix) were removed (http://www.affymetrix.com). The variance of the feature expression values on the PC3 cell lines was used to gauge the technical versus biological variability. Features with the highest 10% variance in the PC3 cell lines were removed from the expression matrix. Lastly, in order to evaluate and remove batch effect, the data was decomposed into its principal components and an analysis of variance model was used. As suggested by a previous study [36], the first 10 principal components were examined for their correlation with batch effect. From these 10 principal components (capturing 31% of the total variance), the two components that were most highly correlated with batch effect were removed.

### Definition of Training and Validation Sets, Feature Selection and Genomic Classifier Development

#### Training and validation sets

After assessing the molecular differences among the three patient groups, very limited differential expression was observed between the NED and PSA-recurrence groups. Differential expression of individual features was obtained through pairwise comparisons of the outcome groups (Crisan et al., manuscript in preparation). At a fold-change threshold of 1.5 (after correcting for false-discovery), only 2 (out of ∼1.4 million) features were found to be differentially expressed between NED and PSA groups, compared to 1186 and 887 in metastasis outcomes compared to NED and BCR-only groups, respectively [37]. Therefore, and in order to develop a signature that predicts early clinical metastasis, these two groups were combined into a single control group. The assignment of patients into training (n = 359) and validation (n = 186) was as defined in our previous study [20].

#### Feature selection

Given the initially large number of features (∼1.4 million), each feature was filtered using a t-test (p<0.01) for complexity reduction on the training set (Figure S1). Features were further vetted in subsequent selection steps. To identify robust features, regularized logistic regression was applied [38], [39] with an elastic net penalty of α = 0.5. This procedure was bootstrapped 1,000 times and the number of times a feature was selected by the regularized regression was tallied. Features that were selected at least 25% of the time were used for classifier development.

#### Genomic classifier development

A random forest machine learning algorithm was used to assemble the selected features into a classifier [40]. A final selection step was used to optimize the feature set on the classification algorithm. Using the rfcv function within the randomForest package [41], the 10-fold cross validation mean squared error (MSE) of models with decreasing numbers of features was plotted. In each iteration, features were excluded if they had the lowest 10% Gini Index. Features that showed little contribution to the performance of the model were not included in the final classifier, keeping those features above the knee of the MSE curve (Figure S2). With this final feature set, the mtry and nodesize random forest parameters were tuned with an accuracy-optimizing grid search. The search of the parameter space was pursued with the tune.randomForest function in the e1071 package [42]. Specifically, the training set (composed of 359 samples) was further split into 1/3 training and 2/3 testing and used with 1000 iterations of bootstrapping to improve performance estimates and control over-fitting. The final genomic classifier (GC) outputs a continuous variable score ranging between 0 and 1, where a higher score indicates a higher probability of clinical metastasis.

#### Clinical classifier and integrated genomic clinical classifier

To benchmark the prognostic ability of GC, we developed a ‘clinical-only’ classifier (CC), trained on the same patients used to discover GC. CC combines pathologic GS, pre-operative PSA (pPSA), SM+, SVI, ECE and N+ using logistic regression. When scoring patients, CC produces a score between 0 and 1, analogous to GC. Additionally, in order to measure the joint prognostic ability of the molecular signature and clinicopathologic variables, an integrated genomic-clinical classifier (GCC) was constructed by combining the CC and GC models using logistic regression.

### Comparison Against External Biomarker Signatures

The performance of GC was compared to that of previously published gene signatures [11]–[13], [15], [16], [18]–[24], [28]–[30] and individual genomic markers associated with prostate cancer progression including CHGA [43], DAB2IP [44], GOLPH2 [45], PAP [46], ETV1 and ERG [47], KI-67 [48], PSA [49], PSCA [50], PSMA [51], AMACR [52], GSTP1 [53], PCA3 [54], B7-H3 [55], TOP2A [14] and CAV1 [56]. Each genomic marker and gene in the signatures were mapped to its associated Affymetrix *core* transcript cluster (http://www.affymetrix.com/analysis/index.affx) where available, otherwise the *extended* transcript cluster was used. Based on the fRMA summarized expression values for the individual genes, the signatures were modeled in the training set using a random forest and tuned with the *tune.randomForest* function from the e1071 R package. Tuning involved performing a 20 by 20 grid search to find the optimal “mtry” and “nodesize” model parameters evaluated via 5-fold cross validation in order to maximize accuracy.

### Performance Assessment of Classifiers and Clinical Variables

Statistical analyses were performed in R v2.14.1, and all tests were two-sided using a 5% significance level. The prognostic ability of all classifiers (GC, CC, GCC, and the external biomarker signatures) were compared using area under ROC curves (AUC), discrimination boxplots and univariable (UVA) logistic regression. Importance of the classifiers relative to clinical information and independent prognostic ability were compared using multivariable (MVA) logistic regression.

Clinical variables were calculated, categorized or transformed as follows. GS was dichotomized into groups with the threshold of ≥8; although convention is to segregate GS into three groups (≤6, 7, ≥8) the relative lack of patients with GS≤6 prompted the dichotomization of GS. The pPSA, measured immediately prior to RP, was log_2_-transformed. The following variables were binary: ECE, SVI, SM+, and N+. Hormone and radiation therapy were included as separate binary covariates if administered in an adjuvant (<90 days post-RP) or salvage (following PSA rise) setting. Treatments administered subsequent to clinical metastasis were not included.

Based on a majority rule criterion, the patients with GC, CC and GCC scores greater than 0.5 were classified as high risk whereas those with a score lower or equal than 0.5 were classified as low risk. Kaplan Meier survival curves were generated for the prostate cancer specific mortality (PCSM) and overall survival endpoints. Lastly, all follow-up times were reported using the method described by Korn [57].

## Results

### Clinical Characteristics of Study Population

From the study population of 639 patients [20], 545 (85%) corresponding to 192 cases and 353 controls had available RNA and were successfully hybridized to microarrays for analysis (see methods). The median age of men in this study is 66 (IQR: 61–70) years, with a median of 16.9 years follow-up. The clinical characteristics of these patients are described in Table 1. Overall, 60% of cases (116/192) had GS ≥8 with only six GS ≤6, whereas controls were predominantly GS 7 (57%) and GS ≤6 (16%). A similar proportion of both cases and controls, (49% and 45%, respectively) were pathological stage T3/4. Controls had 47% T2 disease (in contrast to 27% for cases), and 23% of cases were N+, in contrast to just 8% for controls. A slightly higher rate of SM+ was observed in the cases (54%) in comparison to controls (46%). As expected given the study design, the median time to BCR was very similar between the cases (2.3 years) and PSA controls (1.7 years). While there were 21 clinical metastasis events among controls, these occurred with a median of 9.39 (IQR: 7.5–10.95) years, whereas cases experienced much more rapid events with a median of 5.47 (IQR: 3.7–8.14) years post-RP. Overall, the median time to PCSM (n = 132) was 10.5 years. In order to characterize the true biological potential of tumors from patients who progress early to clinical metastasis after rising PSA, we performed transcriptome-wide differential expression analysis to test the hypothesis that an expression signature in primary tumors could better predict clinical metastasis than clinical variables alone.

**Table 1:** Clinical characteristics of cases and controls among training and validation sets.

	Training					Validation			
		Cases		Controls			Cases	Controls	
	Total	Metastasis		PSA	NED	Total	Metastasis	PSA	NED
	n	n (row %)		n (row %)	n (row %)	n	n (row %)	n (row %)	n (row %)
**Study Cohort**	359	129 (36)		121 (34)	109(30)	186	63 (34)	63 (34)	60 (32)
**Pathological Stage**									
pT2N0M0	145	36 (25)		46 (32)	63 (43)	74	16 (22)	31 (42)	27 (36)
pT3/4N0M0	168	60 (36)		70 (42)	38 (22)	85	35 (41)	28 (33)	22 (26)
pTanyN+M0	46	33 (72)		5 (11)	8 (17)	27	12 (44)	4 (15)	11 (41)
**Pathologic Gleason Score**									
≤6	45	4 (9)		18 (40)	23 (51)	18	2 (11)	9 (50)	7 (39)
7	174	44 (25)		70 (40)	60 (35)	97	26 (27)	29 (30)	42 (43)
8	45	17 (38)		16 (35)	12 (27)	23	10 (43)	9 (40)	4 (17)
9	87	57 (65)		17 (20)	13 (15)	47	25 (53)	15 (32)	7 (15)
10	8	7 (87)		0	1 (13)	1	0	1 (100)	0
**Pre-operative Prostate-specific Antigen**									
<10 ng/mL	191	74 (39)		55 (29)	62 (32)	92	32 (35)	29 (31)	31 (34)
10–20 ng/mL	83	21 (25)		33 (40)	29 (35)	33	10 (31)	12 (36)	11 (33)
>20 ng/mL	81	12 (14)		32 (40)	37 (46)	50	16 (32)	18 (36)	16 (32)
Not available	4	1 (25)		1 (25)	2 (50)	11	5 (46)	4 (36)	2 (18)
**Seminal Vesicle Invasion**									
Present	110	56 (51)		35 (32)	19 (17)	66	31 (47)	19 (28)	16 (25)
**Surgical Margin Status**									
Positive	179	73 (40)		60 (34)	46 (26)	87	30 (34)	34 (40)	23 (26)
**Extra-capsular Extension**									
Present	182	80 (44)		63 (35)	39 (21)	91	38 (41)	26 (29)	27 (30)
**Biochemical recurrence**									
Event	260	129 (50)		121 (46)	10 (4.0)	128	63 (49)	63 (49)	2 (2.0)
**Clinical Metastasis**									
Event	143	129 (90)		14 (10)	0	69	62 (90)	7 (10)	0
**Prostate Cancer-specific Mortality**									
Event	96	86 (90)		10 (10)	0	36	32 (89)	4 (11)	0
**Adjuvant Radiation**									
Administered	36	18 (50)		5 (14)	13 (36)	18	10 (56)	4 (22)	4 (22)
**Adjuvant Androgen Deprivation Therapy**									
Administered	77	44 (57)		13 (17)	20 (26)	47	18 (39)	9 (19)	20 (42)
**Salvage Radiation**									
Administered	57	23 (40)		34 (60)	0	25	10 (40)	15 (60)	0
**Salvage Androgen Deprivation Therapy**									
Administered	119	67 (56)		52 (44)	0	53	23 (43)	30 (57)	0

### Development of Models to Predict Early Clinical Metastasis

**Figure 1:**
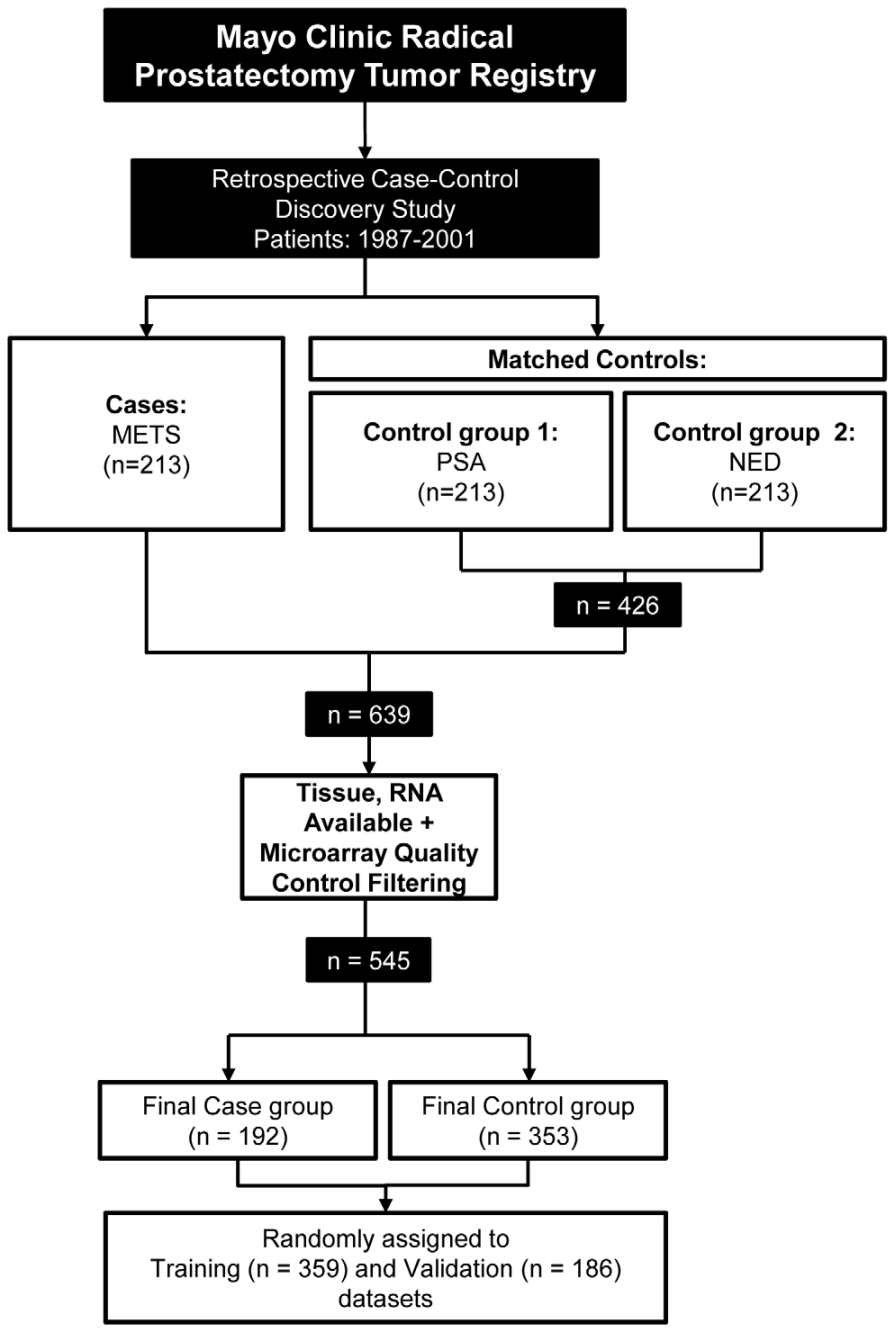
Consort diagram. Study breakdown into cases and controls. Training and validation sets are shown.

**Figure 2:**
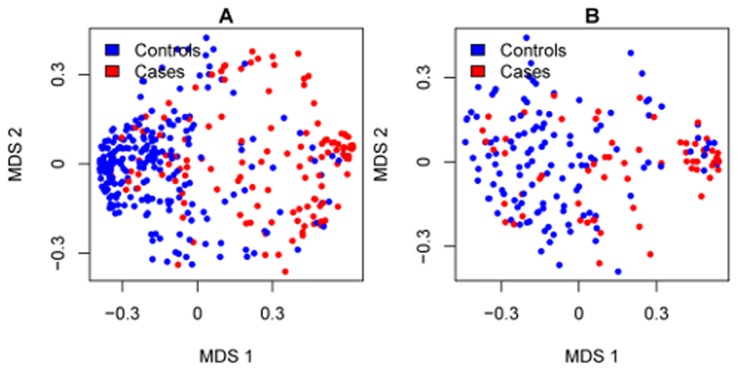
Multidimensional scaling plot of (A) the training and (B) the validation sets. Controls are indicated in blue and cases in red. In both the training and validation sets the controls tend to cluster on the left of the plot and the cases on the right of the plot. In this manner, most of the biological differences are expressed in the first dimension of the scaling. Random forest proximity [http://www.stat.berkeley.edu/~breiman/] was used to measure the 22 marker distance between samples.

Cases and controls were compared and used for the development of a genomic (GC), clinical-only (CC) and integrated (GCC) classifier models for predicting cases (i.e., early clinical metastasis after rising PSA) as the primary endpoint (see methods). The 545 samples were assigned to training (n = 359, 39% cases) and validation (n = 186, 37% cases) sets (Figure 1). GC was developed from analysis of 1.1 million RNA features on the microarray in the training set after removal of cross-hybridizing and unreliable features (see methods). An initial feature selection step based on t-tests for complexity reduction yielded 18,902 differentially expressed features between cases and controls (Figure S1). Further selection of these differentially expressed features by regularized logistic regression reduced the list to a total of 43. As a final step, these 43 differentially expressed features were further filtered to only those that demonstrated to improve a random forest-based performance metric (see methods). This resulted in a final set of 22 markers corresponding to RNAs from coding and non-protein coding regions of the genome (Table 2). Multidimensional scaling analysis depicts clustering of cases and controls based on expression of the 22 markers (Figure 2). A random forest machine-learning algorithm was used to generate GC scores after assembling the 22 markers with forest parameters to optimize for highest accuracy in the training set. Logistic regression was used to assemble the six clinicopathologic risk factors into a CC and also integrated with GC to build a GCC.

**Table 2:** Summary description of the 22 markers in the genomic classifier.

Marker	NearestGene/Locus	Type of Marker	Cytoband	AndrogenRegulated^1^	Biological Process(es)	Reference(s) _[PMID]_
1	*LASP1*	CODING	17q12		Cell Proliferation, Differentiation	Grunewald et al, 2007 _[17211471]_; Traenka et al, 2010 _[20924110]_
2	*IQGAP3*	3' UTR	1q23.1		Cell Proliferation, Differentiation	Nojima et al, 2008 _[18604197]_
3	*NFIB*	INTRONIC	9p23		Cell Proliferation, Differentiation	Qian et al, 1995 _[7590749]_; Dooley et al, 2011 _[21764851]_
4	*S1PR4*	3' UTR	19p13.3		Cell Proliferation, Differentiation	Yamazaki et al, 2000 _[10679247]_
5	*THBS2*	3' UTR	6q27		Cell Structure, Adhesion, Motility	Volpert et al, 1995 _[8526929]_; Kyriakides et al, 2001 _[11583953]_
6	*ANO7*	3' UTR	2q37.3	Yes	Cell Structure, Adhesion, Motility	Das et al, 2008 _[18676855]_
7		NON-CODING TRANSCRIPT*				
8	*PCDH7*	INTRONIC	4p15.1	Yes	Cell Structure, Adhesion, Motility	Yoshida, 2003 _[12949613]_
9	*MYBPC1*	CODING	12q23.2	Yes	Cell Structure, Adhesion, Motility	Gregg et al, 2010 _[20426842]_
10		INTRONIC				
11	*EPPK1*	3' UTR	8q24.3	Yes	Cell Structure, Adhesion, Motility	Yoshida et al, 2008 _[18498355]_
12	*TSBP*	INTRONIC	6p21.32		Immune Response	Liang et al, 1994 _[7530381]_
13	*PBX1*	CODING	1q23.3	Yes	Immune Response	Chung et al, 2007 _[18093541]_; Kikugawa et al, 2006 _[16637071]_; Qiu et al, 2007 _[17200190]_
14	*NUSAP1*	3' UTR	15q15.1		Cell Cycle Progression, Mitosis	Raemaekers et al, 2003 _[12963707]_; Ribbeck et al, 2007 _[17276916]_
15	*ZWILCH*	3' UTR	15q22.31		Cell Cycle Progression, Mitosis	Williams et al, 2003 _[12686595]_
16	*UBE2C*	3′UTR	20q13.12	Yes	Cell Cycle Progression, Mitosis	Rape and Kirschner, 2004 _[15558010]_
17		CODING ANTISENSE				
18	*CAMK2N1*	CODING ANTISENSE	1p36.12	Yes	Cell Cycle Progression, Mitosis	Wang et al, 2008 _[18305109]_
19	*RABGAP1*	EXON/INTRON JUNCTION ANTISENSE	9q33.2		Cell Cycle Progression, Mitosis	Cuif et al, 1999 _[10202141]_
20	*PCAT*-*32*	NON-CODING TRANSCRIPT	5p15.2		Other, Unknown Function	Prensner et al, 2011 _[21804560]_
21	*GLYATL1P4*/*PCAT*-*80*	NON-CODING TRANSCRIPT	11q12.1		Other, Unknown Function	Prensner et al, 2011 _[21804560]_
22	*TNFRSF19*	INTRONIC	13q12.12		Other, Unknown Function	Eby et al, 2000 _[10809768]_

* Overlaps with an exon of a 'retained intron' category.

1 Based on Jiang et al. Mol Endocrinol 23∶1927-33, 2009; Massie et al. EMBO Rep 8∶871-8, 2007.

### Classifier Performance in Training and Validation Set

**Figure 3:**
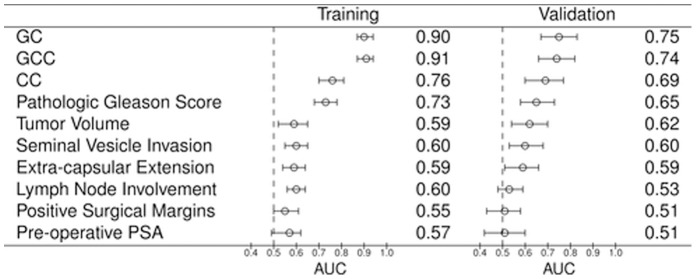
Performance of classifiers and individual clinicopathologic variables. For each predictor, the AUC obtained in the training and validation sets, as well as the 95% Confidence Interval for this metric is shown. CC: clinical-only classifier. GC: genomic classifier. GCC: combined genomic-clinical classifier.

**Figure 4:**
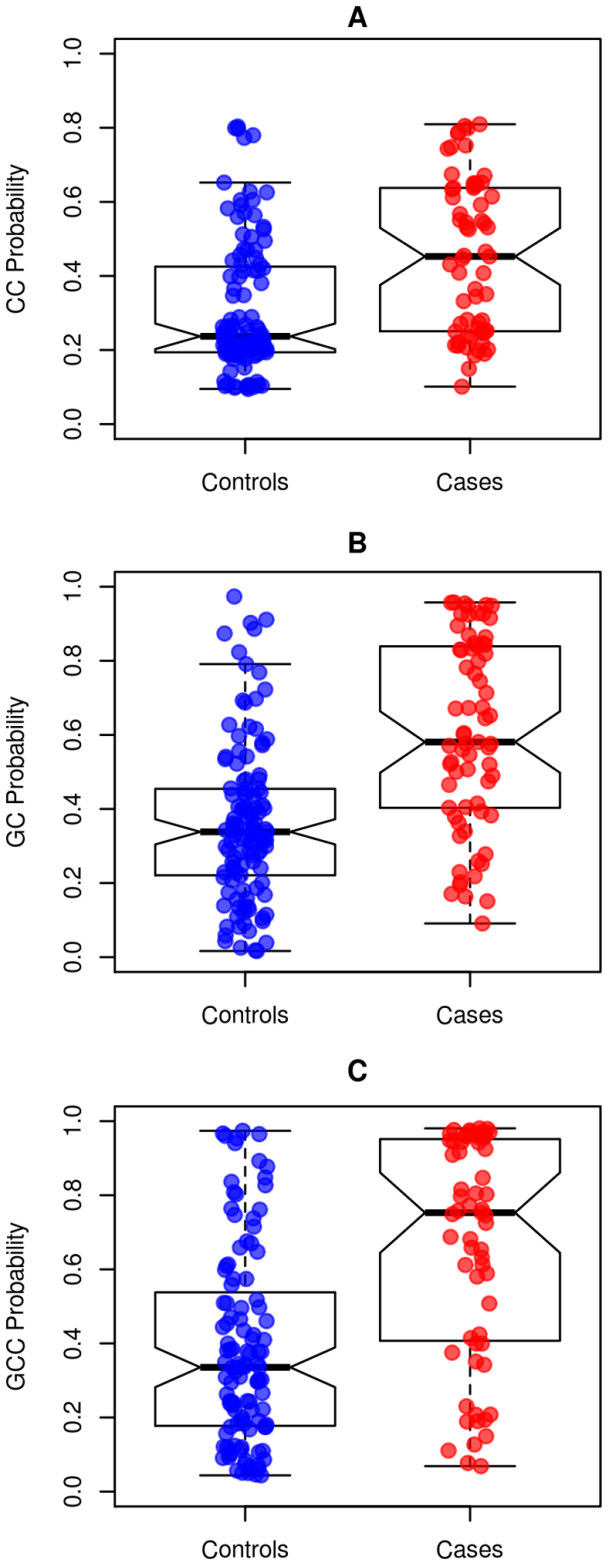
Score distributions of multivariable classifiers in cases and controls in validation set. Distributions of scores are plotted for A) CC B) GC and C) GCC for controls and cases. Median scores and 95% confidence intervals are represented by a horizontal black line and notches, respectively. Non-overlapping notches indicate that differences in the distribution of scores between cases and controls are statistically significant. Outliers are represented as points beyond the boxplot whiskers.

In the training set, ROC area-under the curve (AUC) values for GC, CC and GCC were 0.90, 0.76 and 0.91 respectively, higher than any individual clinical variable (Figure 3). In the validation set, GC and GCC had the highest AUC of 0.75, and 0.74, respectively for predicting cases. The clinical-only CC had an AUC of 0.69, which was only marginally better than pathological GS alone (0.65). The shape of the ROC curves for GC and GCC shows that these models have the highest specificity and sensitivity compared to clinical models above a threshold of ∼50% specificity (Figure S3). Discrimination box plots further show greater median differences in GC and GCC scores between cases and controls than for CC (Figure 4).

### GC Reclassification of GS Groups

**Figure 5:**
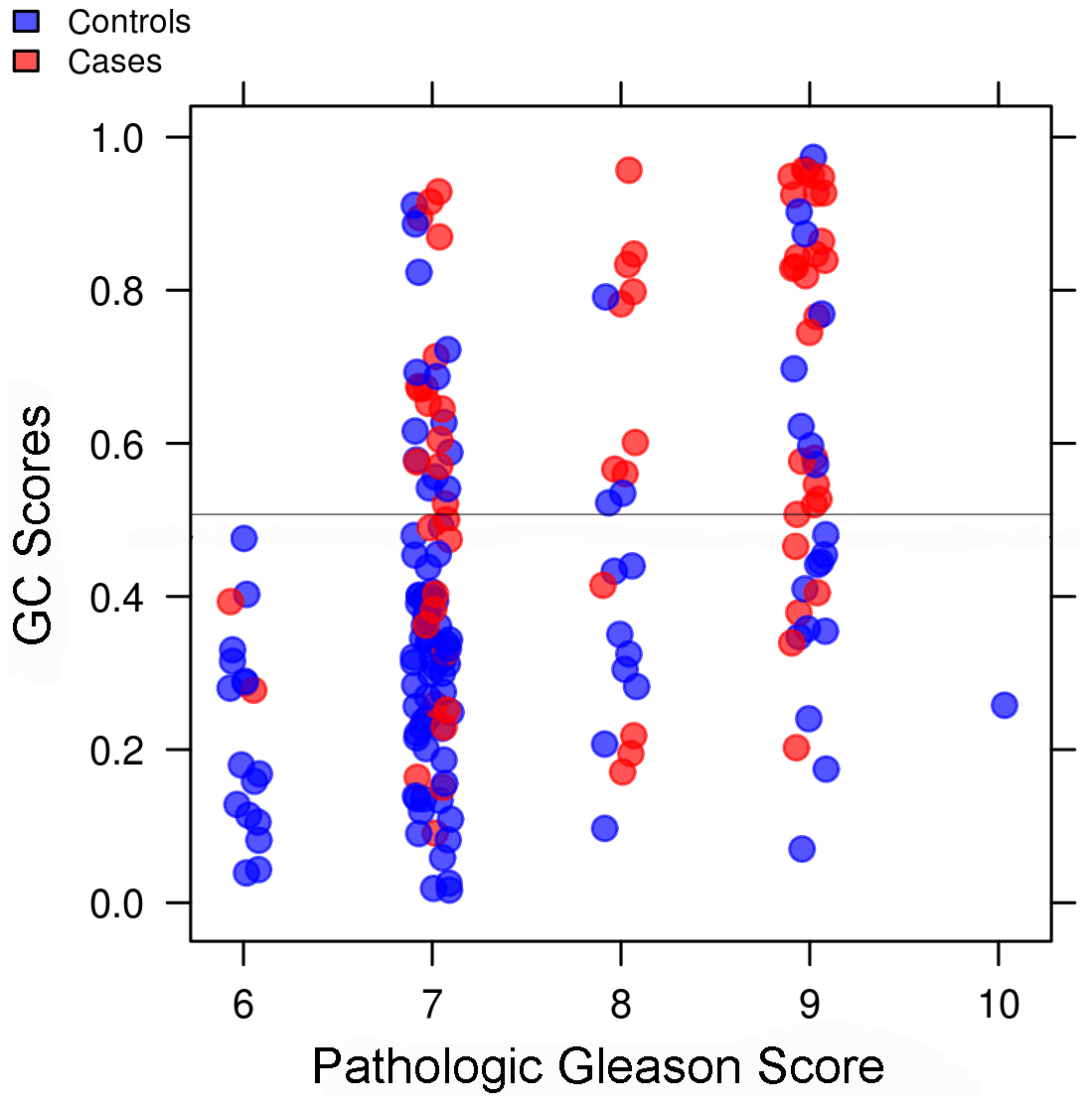
Distribution of GC scores among pathologic GS categories in validation. GC scores are plotted with a jitter so as to more easily differentiate the patients among each pathologic GS (x-axis) groups. Case (red) and controls patients (blue) are shown for each category. The dashed black line indicates the GC cutoff of 0.5. Trends show the patients with high GC scores tend to have high GS as well.

The distribution of cases and controls in the validation set by both GC and GS [58] risk groups is illustrated in Figure 5 and summarized in Table 3. Among GS ≤6 tumors (n = 18) none had high GC scores, while among GS 7 tumors (n = 97), nearly a third (29%) had high GC scores and half of these were cases that developed early metastasis after rising PSA. While most patients with high GS (≥8) had high GC scores, among the 29 (40%) with low GC scores there were only 7 cases with 3 deaths from prostate cancer. Overall, 116 out of 186 (62%) validation set patients had low GC scores of which only 21 were cases resulting in 7 deaths from prostate cancer. Among the 70 (38%) patients with high GC scores, there were 42 cases and 25 of these men died of prostate cancer.

**Table 3:** Reclassification by GC of GS risk categories among cases and controls in the validation set of patients.

	GC ≤0.5			GC >0.5		
Gleason Category	n	n METs(%)	n PCSM(%)	n	n METs(%)	n PCSM(%)
GS ≤6	18	2 (11)	0	0	0	0
GS 7	69	12 (17)	4 (5.7)	28	14 (50)	4 (14)
GS 8	12	4 (33)	1 (8.3)	11	6 (54)	5 (45)
GS ≥9	17	3 (17)	2 (12)	31	22 (70)	16 (51)

Pathologic GS is categorized into four groups: ≤6,7, 8 and ≥9. Gleason groups are re-classified by high (>0.5) and low GC risk scores. Total number of patients in each category is further subdivided into the number of cases and those that died of prostate cancer (PCSM).

### GC is an Independent Prognostic Variable

In order to test for the effect size of individual variables as well as dependencies among these variables we performed univariable and multivariable analyses using logistic regression on the validation set (Table 4). In univariable analysis, we found GC, CC, GCC, GS, SVI and ECE to be statistically significant predictors of cases (p<0.05). The odds ratio for GC was 1.42 for every 10% increase in GC score. When dichotomized into low and high GC risk groups, as described above, the odds ratio was 6.79 (95% CI: 3.46–13.29), more than twice the odds ratio of GS (OR: 3.02 (95% CI: 1.61–5.68)) for predicting cases. In multivariable analysis, after adjustment for post-RP treatment, GC remained the only significant prognostic variable (p<0.001) with an OR of 1.36 for every 10% increase in GC score. The independent significance of GC suggests that a more direct measure of tumor biology (i.e., 22-marker expression signature) adds significant prognostic information for prediction of early metastasis after rising PSA, which is not captured by the clinical variables available from pathological analysis.

**Table 4:** Univariable and multivariable odds Ratios for CC, GC and GCC, and clinicopathologic variables.

	Univariable		Multivariable	
	Odds Ratio (95% CI)	P	Odds Ratio (95% CI)	P
GC	1.42 (1.28–1.60)	p<0.001	1.36 (1.16–1.60)	p<0.001
GCC	1.36 (1.21–1.53)	p<0.001	n.a	n.a
CC	1.35 (1.15–1.59)	p<0.001	n.a	n.a
Pre-operative PSA	0.99 (0.77–1.26)	0.92	0.75 (0.52–1.07)	0.11
Pathologic Gleason Score ≥8	3.02 (1.61–5.68)	p<0.001	1.91 (0.85–4.33)	0.12
Seminal Vesicle Invasion	2.44 (1.30–4.58)	0.01	1.93 (0.79–4.73)	0.15
Tumor Volume	1.02 (0.97–1.06)	0.44	0.97 (0.92–1.04)	0.42
Lymph Node Involvement	1.69 (0.74–3.88)	0.21	1.42 (0.41–4.96)	0.58
Positive Surgical Margins	1.05 (0.57–1.93)	0.87	0.93 (0.40–2.17)	0.87
Extra-capsular Extension	2.01 (1.18–3.73)	0.03	1.00 (0.45–2.20)	0.99

Odd ratios for multivariable classifiers are adjusted as indicated in the Materials and Methods. CC: clinical-only classifier. GC: genomic classifier. GCC: integrated genomic-clinical classifier.

### Cases with High GC Scores Die Earlier from Prostate Cancer and other Causes

**Figure 6:**
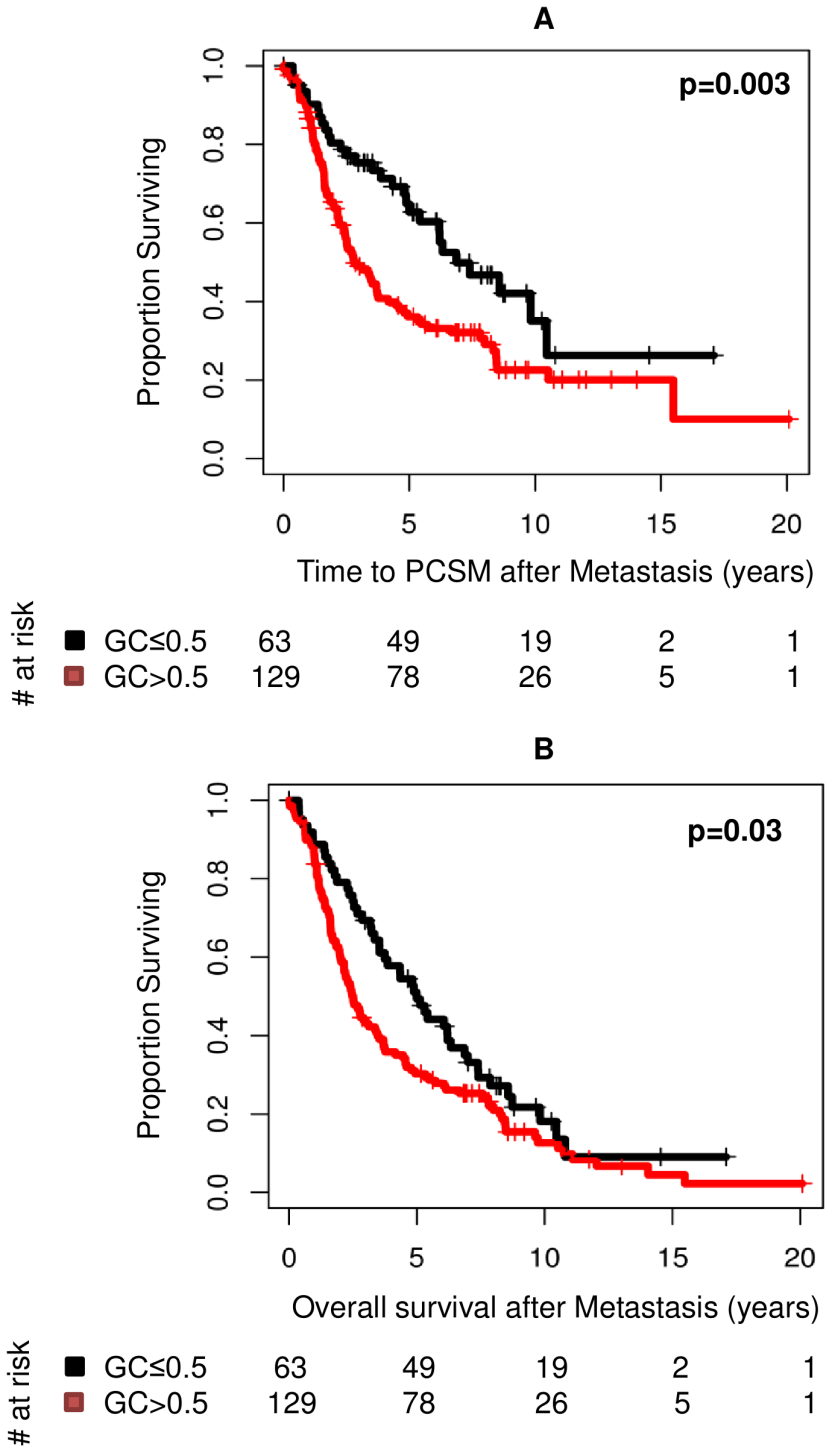
Kaplan Meier estimates for all Cases with (A) PCSM and (B) OS endpoints. Cases were separated into high (>0.5) or low risk according to GC score. Log-rank p-values are shown in the upper right corner. Time to PCSM and OS is measured from BCR in years.

We next compared the survival outcomes of cases and controls in Kaplan-Meier analysis of low and high GC score groups. Cases with lower GC scores had a median 6.9 year prostate-cancer specific survival compared to median 2.9 years for cases with high GC scores (p = 0.003) (Figure 6). For overall survival, there was a significant (p = 0.03) difference in outcome, with median overall survival after metastasis of 2.5 and 4.98 years for cases with high and low GC scores, respectively. Among all controls, 21 patients developed clinical metastasis outside of the study case-control definitions (i.e. >5 years after rising PSA). We evaluated whether GC was able to segregate patients that had late occurring metastasis events among the PSA controls (Figure S4). GC was able to significantly (p<0.05) separate those PSA patients that would go on to experience later clinical metastasis, from those that did not. This difference in outcomes further strengthens the notion that GC measures a component of the biological potential for metastasis and that those patients with the highest GC scores may be most at risk for early metastatic progression post-RP.

### Comparisons to External Biomarker Signatures

**Figure 7:**
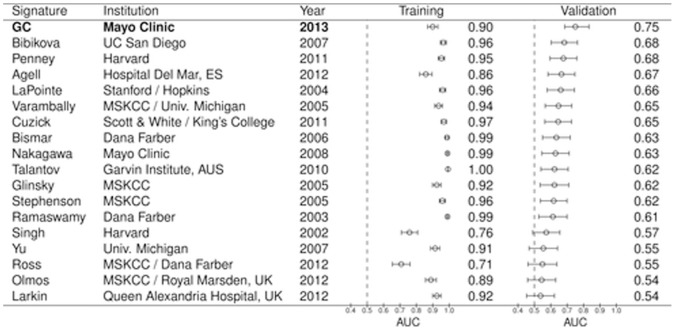
Performance of external signatures in training and validation sets. For each signature, the institution associated to it, year of publication, lead author, the AUC obtained in the training and validation sets, as well as the 95% Confidence Interval for this metric is shown.

In order to compare the performance of GC to previously reported gene signatures, we compiled the genes associated to external signatures and combined them into a Random Forest classifier (see methods). In addition, we evaluated the expression of individual genes previously reported to be associated to prostate cancer outcomes. The performance of the classifiers and the individual genes was subsequently assessed in both training and validation sets (Figures 7 and S5). As expected, we observe high AUCs in training for nearly all the external signatures, similar to what was observed with GC. When applied to validation, the AUC for each model decreased. Among the 17 external signatures that were modeled, 12 were statistically significant predictors of metastasis (i.e., their 95% confidence intervals did not drop below a threshold random chance AUC of 0.5) (Figure 7). The AUC of GC was 0.08 points higher than the top performing external signature, the 16-gene signature reported by Bibikova et al [12], which had an AUC of 0.68 (95% CI : 0.60–0.76,). In contrast to the expression signature models, the performance of the 16 single genes tested were expected to be similar in the training and validation sets. These genomic markers show an overall agreement in performance, with differences in significance likely explained by the smaller sample size of the validation set compared to the training set (Figure S5). Of the 16 genomic markers, only B7-H3 (CD276), GSTP1 and PCA3 were statistically significant in both the training and validation sets (Figure S5). Again, none of the individual genomic markers outperform GC or the top performing clinical predictor, GS (AUCs ≤0.64).

## Discussion

This study was designed to test the hypothesis that biological assessment of both coding and non-coding expression profiles in primary tumors could predict the development of early clinical metastasis following BCR. We discovered a 22-marker genomic classifier (GC) that, without sacrifice of sensitivity, was more specific in validation than established prognostic factors such as GS. Based on the results presented here, GC measures a component of the biologic potential for early clinical metastasis better than clinical variables or previously reported biomarker signatures. This may enable clinicians to better select the best candidates for intensive multi-modal therapy and spare those not at risk the morbidity of post-RP interventions.

Here we profile the expression of over 1.4 million RNA features in FFPE primary tumor specimens from 545 patients, of whom 192 developed early clinical metastasis, representing to our knowledge the largest high-resolution genomic discovery and validation effort of aggressive prostate cancer to date. The long term follow-up (median 16.9 years) allowed us to evaluate GC for more definitive endpoints such as clinical metastasis and prostate cancer specific mortality compared to previous biomarker studies that focused on surrogates such as Gleason grade or biochemical recurrence (e.g. [11], [15]). We benchmarked the improved performance of GC against individual clinical factors and multivariable clinical risk models as well as previously reported single and multi-marker expression signatures. While GC outperforms the previously reported signatures and individual markers, we acknowledge that differences in methodology, study design, and endpoint may impact performance of these signatures and biomarkers. To avoid over-fitting bias skewed in favor of GC, we retrained the previously reported multi-marker expression signatures (e.g., Cuzick et al [15], CCP) in the training set. In validation, GC outperformed all individual variables including GS, clinicopathologic features and single biomarkers (e.g., KI-67, TOP2A) and the clinical-only multivariable classifier (CC). CC was integrated with GC into a genomic-clinical classifier (GCC) and we observed that the genomic features contributed the bulk of prognostic information upon multivariable analysis, with GCC having the same prognostic abilities as GC.

The high-density array used in this study permits measurement of the expression patterns of RNAs associated with multiple biological processes in prostate cancer progression. The biological processes represented in the GC signature include cell cycle progression, cell adhesion, tumor cell motility, migration and immune system modulation (see Table 2). Furthermore, many of the genes have evidence of being involved in androgen signaling. For example, MYBPC1, UBE2C and NUSAP1 have been previously reported to be differentially expressed throughout prostate cancer progression [22]. Differential expression analysis between androgen-dependent and androgen-independent cell lines [59] found the protocadherin gene PCDH7 to have the largest fold change, suggesting it may play a role in the development of castrate-resistant prostate cancer. Thrombospondin-2, a modulator of angiogenesis, has also been reported to be differentially expressed when comparing non-metastatic and metastatic prostate cancer samples in two independent studies [60], [61]. Also, the cytoskeleton associated genes EPPK1, a plakin family member, and the LIM and SH3 protein gene LASP1 fall in regions 8q24 and 17q12; the gains of both regions have been previously associated with prostate cancer progression [62], [63]. Additionally, LASP1 is a target of the microRNA MIR-203, a gene known to control proliferation, migration, and invasive potential of prostate cancer cell lines [64]. ANO7, also known as NGEP (for New Gene Expressed in Prostate) is an androgen-dependent gene known to be specifically expressed in epithelial cells of prostate cancer and in normal prostate, but not in other tissues [65]. Furthermore, this gene has been regarded as a target for antibody-based immunotherapy [65]. Interestingly, two of the genes, PBX1 and TSBP are linked to immune system regulation. In the case of PBX1, a previous study has shown that this gene transcriptionally regulates the immunoregulatory cytokine IL10 by binding to the apoptotic cell response element of this gene [66]. The genomic locus containing gene TSBP (also known as C6orf10) is located in the classical Class II block of the Major Histocompatibility Complex (MHC) region in chromosome 6 [67], [68].

Two components of this signature correspond to previously reported long ncRNAs differentially expressed in prostate cancer: Prostate Cancer Associated Transcript (PCAT) 32 and PCAT-80 [33]. PCAT-80 largely overlaps with a pseudogene known as GLYATL1P4 (for glycine N-acyltransferase-like protein 1 pseudogene 4). The functional version of this pseudogene, GLYATL1, has been found to be differentially expressed in a cell line-based prostate cancer progression model [69]. Furthermore, this gene encodes an enzyme that is associated to N-Acetyl Glutamic Acid, a metabolite found at abnormal concentrations in urine in prostate cancer (HMDB01138 in the Human Metabolome Database, [70], [71]). These results on GLYATL1P4 and GLYATL1 provide further evidence that pseudogenes may play a role in prostate cancer progression and may be functionally associated with their coding mRNA partners [72]. Other sources of evidence including lncRNAs known to be involved in prostate cancer adjacent to genes comprising GC (e.g. PCAT-113 [33], found 200 bps upstream of CAMK2N1) as well as overlapping copy number alterations found in prostate tumors (e.g. the copy number amplification reported by Taylor et al [73] in chr5p15.2 and PCAT-32) add to the sources of evidence on their association with prostate cancer and involvement in multiple biological processes that must occur for tumorous tissue to leave the prostate bed during the metastatic process.

Several RNA components of GC correspond to transcription units within intronic regions or to the anti-sense version of a given gene. Detailed studies to elucidate the functional role of these specific RNAs have not been published. These RNA features may belong to a different transcriptional unit than currently annotated. Additional experimental validation and assessment of the RNAs included within GC will shed further light on their biology and their specific roles in prostate cancer progression.

When associations of GC with pathologic GS - the most prognostic clinicopathologic variable - were examined, we observed that most patients with high GC scores had high pathologic GS, and many experienced clinical metastasis and prostate cancer specific mortality. However, GC is able to re-stratify GS risk groups while retaining high sensitivity for predicting early metastasis after rising PSA, especially in intermediate risk patients with GS 7 tumors. While not all patients with high GC scores experienced metastasis, many of these patients may have been treated more aggressively because they had high-risk pathology, thereby delaying disease progression. Furthermore, this study population received variable treatment regimens as would be expected in a non-randomized institutional cohort. Such differences will have an impact on the development of metastatic and lethal events. In addition, because we used a nested case-control design we could not obtain true metastasis-free survival estimates (as would have been possible with a case-cohort study). Therefore, additional studies including those from randomized controlled clinical trials are necessary to determine whether GC can provide predictive information on benefit or response to treatment. However, our retrospective study suggests that GC will provide predictive information when utilized in such prospective trials.

### Conclusion

We developed a 22-marker genomic classifier containing a large number of non-coding RNA sequences using FFPE tumor tissue specimens obtained from a large cohort of men that had radical prostatectomy for localized prostate cancer. The classifier was validated and showed significantly superior performance in predicting early clinical metastasis compared to previously described individual genes, multigene signatures and clinicopathologic variables. To our knowledge this represents the largest study of prostate cancer patients exploring clinically relevant endpoints using a high-density, transcriptome-wide approach for differential expression analysis. GC offers improved risk stratification among post-RP patients and may better identify patients that require intensive multi-modal therapy, while sparing those who can be closely monitored without initiating aggressive adjuvant treatment. The reassignment of risk groups for patients with different pathological GS based on GC scores indicates that genomic markers presumably measure the biological potential of the tumor to metastasize and can add an additional layer of detail not captured by clinicopathologic variables. GC can be used immediately following RP and, because it can accurately predict metastasis long before it can be detected radiographically, may better guide post-surgical treatment decisions.

## Supporting Information

Figure S1 **Summary of methods of GC development.** Methods are separated based on array summarization, normalization and quality controls (pre-processing) followed by steps used for feature selection and classifier assembly (model building).
(TIFF)

Figure S2 **Example of the mean squared error vs feature set size plot used to reduce the genomic feature set size from 43 to 22 features.** 10 fold cross validation was used to assess the MSE of each random forest model constructed from decreasing feature set sizes. Features were eliminated based on having the lowest variable importance ranked by the Gini index. The vertical dotted line is drawn at the 22 feature mark, where the MSE is minimized and the knee of the plot occurs.
(TIFF)

Figure S3 **ROC curve of multivariable models and clinicopathologic variables.** A) ROC curves in Training B) ROC curves in the validation set.
(TIFF)

Figure S4 **Kaplan Meier estimates for all PSA Controls with metastasis endpoint. PSA** controls were separated into two groups based on high (>0.5) or low risk according to GC. The log-rank p-value is shown in the upper right corner.
(TIFF)

Figure S5 **Performance of single genes in training and validation sets.** For each gene, the AUC obtained in the training and validation sets, as well as the 95% Confidence Interval for this metric is shown.
(TIFF)
