# Supplementary figures and images for: Discovery and Validation of a Prostate Cancer Genomic Classifier that Predicts Early Metastasis Following Radical Prostatectomy
Source: PLoS One. 2013 Jun 24;8(6):e66855. doi: 10.1371/journal.pone.0066855 (PMC3691249; doi:10.1371/journal.pone.0066855)

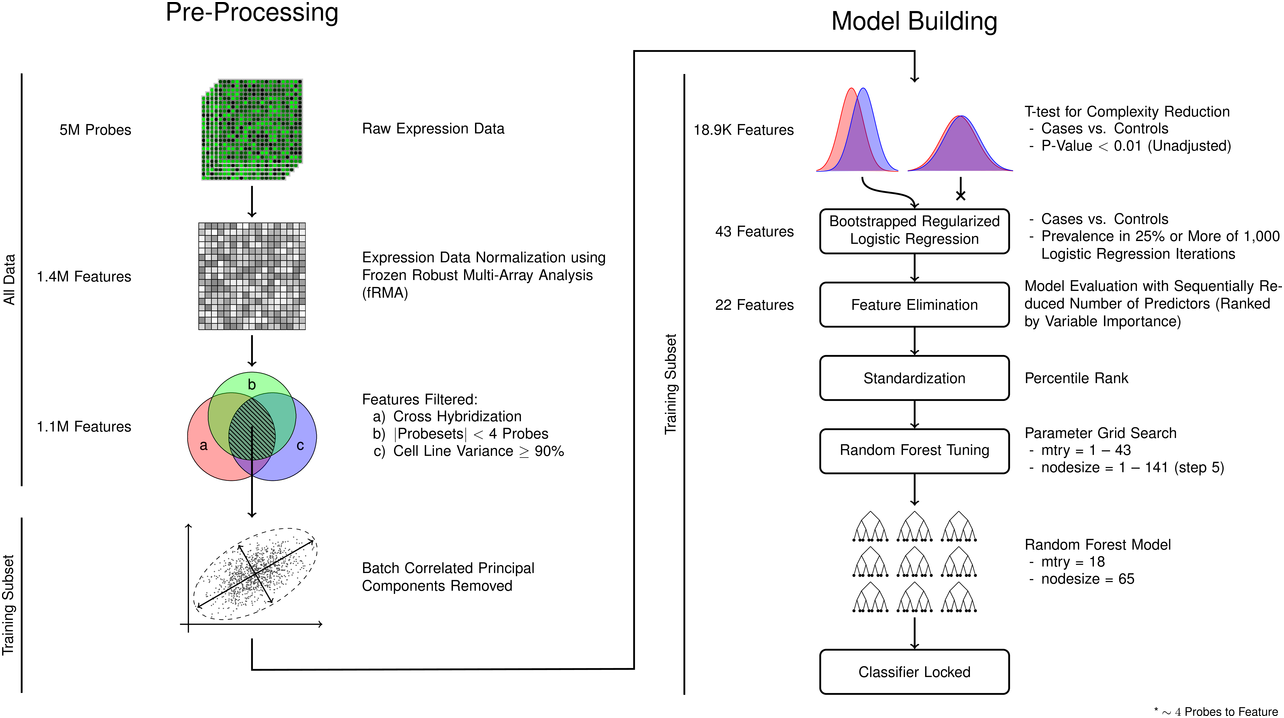

Supplement: Figure S1 — Summary of methods of GC development. Methods are separated based on array summarization, normalization and quality controls (pre-processing) followed by steps used for feature selection and classifier assembly (model building). (TIFF) [file pone.0066855.s001.tiff]

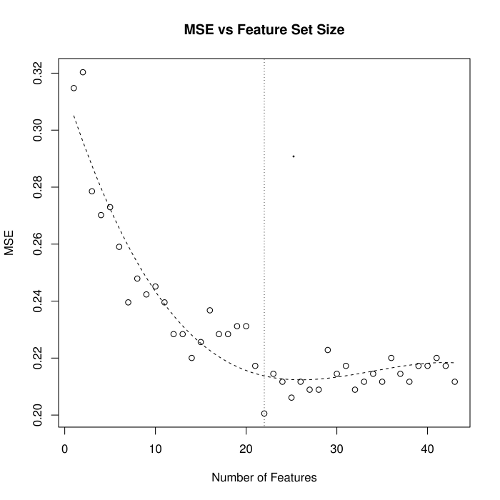

Supplement: Figure S2 — Example of the mean squared error vs feature set size plot used to reduce the genomic feature set size from 43 to 22 features. 10 fold cross validation was used to assess the MSE of each random forest model constructed from decreasing feature set sizes. Features were eliminated based on having the lowest variable importance ranked by the Gini index. The vertical dotted line is drawn at the 22 feature mark, where the MSE is minimized and the knee of the plot occurs. (TIFF) [file pone.0066855.s002.tiff]

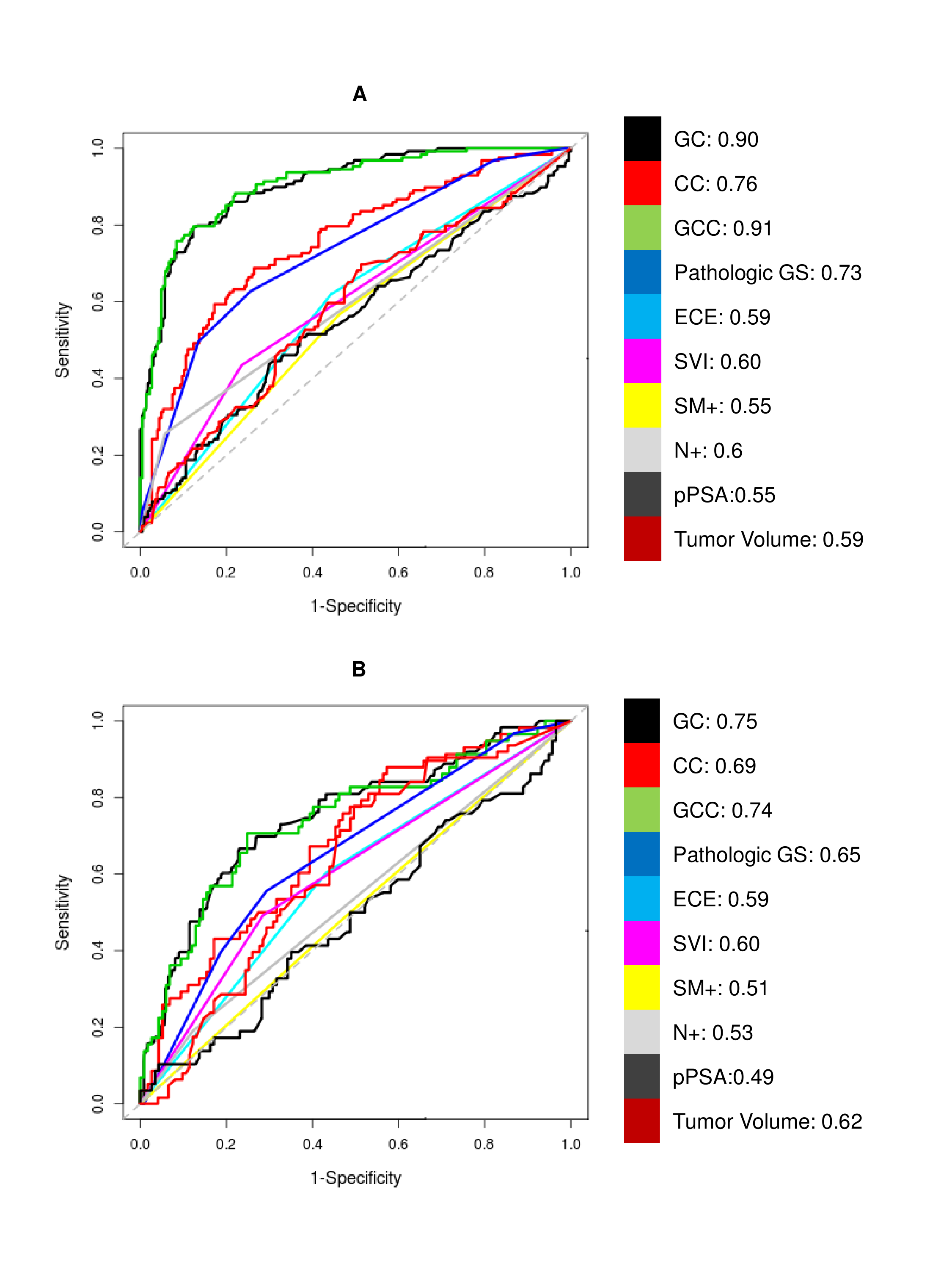

Supplement: Figure S3 — ROC curve of multivariable models and clinicopathologic variables. A) ROC curves in Training B) ROC curves in the validation set. (TIFF) [file pone.0066855.s003.tiff]

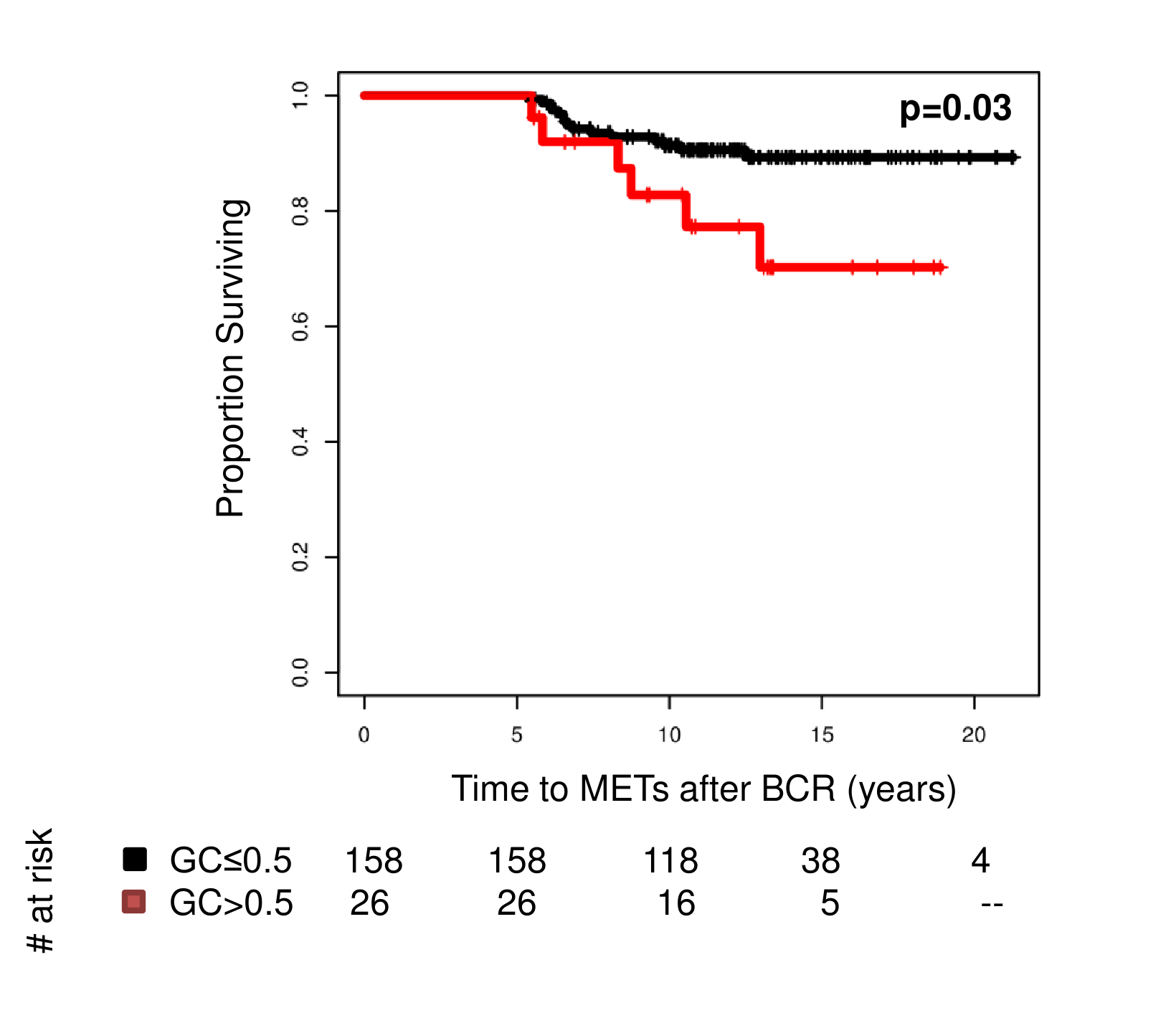

Supplement: Figure S4 — Kaplan Meier estimates for all PSA Controls with metastasis endpoint. PSA controls were separated into two groups based on high (>0.5) or low risk according to GC. The log-rank p-value is shown in the upper right corner. (TIFF) [file pone.0066855.s004.tiff]
